# Endochondral Bone Regeneration by Non-autologous Mesenchymal Stem Cells

**DOI:** 10.3389/fbioe.2020.00651

**Published:** 2020-07-09

**Authors:** Alessia Longoni, I. Pennings, Marta Cuenca Lopera, M. H. P. van Rijen, Victor Peperzak, A. J. W. P. Rosenberg, Riccardo Levato, Debby Gawlitta

**Affiliations:** ^1^Department of Oral and Maxillofacial Surgery & Special Dental Care, University Medical Center Utrecht, Utrecht University, Utrecht, Netherlands; ^2^Regenerative Medicine Center Utrecht, Utrecht, Netherlands; ^3^Laboratory for Translational Immunology, University Medical Center Utrecht, Utrecht, Netherlands; ^4^Department of Orthopaedics, University Medical Center Utrecht, Utrecht University, Utrecht, Netherlands; ^5^Department of Clinical Sciences, Faculty of Veterinary Medicine, Utrecht University, Utrecht, Netherlands

**Keywords:** allograft, xenograft, graft rejection, adaptive and innate immune response, bone regeneration, chondrogenic differentiation, endochondral bone formation, MSCs

## Abstract

Mimicking endochondral bone formation is a promising strategy for bone regeneration. To become a successful therapy, the cell source is a crucial translational aspect. Typically, autologous cells are used. The use of non-autologous mesenchymal stromal cells (MSCs) represents an interesting alternative. Nevertheless, non-autologous, differentiated MSCs may trigger an undesired immune response, hampering bone regeneration. The aim of this study was to unravel the influence of the immune response on endochondral bone regeneration, when using xenogeneic (human) or allogeneic (Dark Agouti) MSCs. To this end, chondrogenically differentiated MSCs embedded in a collagen carrier were implanted in critical size femoral defects of immunocompetent Brown Norway rats. Control groups were included with syngeneic/autologous (Brown Norway) MSCs or a cell-free carrier. The amount of neo-bone formation was proportional to the degree of host-donor relatedness, as no full bridging of the defect was observed in the xenogeneic group whereas 2/8 and 7/7 bridges occurred in the allogeneic and the syngeneic group, respectively. One week post-implantation, the xenogeneic grafts were invaded by pro-inflammatory macrophages, T lymphocytes, which persisted after 12 weeks, and anti-human antibodies were developed. The immune response toward the allogeneic graft was comparable to the one evoked by the syngeneic implants, aside from an increased production of alloantibodies, which might be responsible for the more heterogeneous bone formation. Our results demonstrate for the first time the feasibility of using non-autologous MSC-derived chondrocytes to elicit endochondral bone regeneration *in vivo*. Nevertheless, the pronounced immune response and the limited bone formation observed in the xenogeneic group undermine the clinical relevance of this group. On the contrary, although further research on how to achieve robust bone formation with allogeneic cells is needed, they may represent an alternative to autologous transplantation.

## Introduction

Endochondral bone formation represents a key process in bone development and fracture healing (Gerstenfeld et al., 2003; Shapiro, 2008). In particular, the growth plate and the fracture callus are characterized by a highly organized cartilaginous structure where chondrocytes progressively acquire a hypertrophic phenotype (Gerstenfeld et al., 2003). Once hypertrophic, chondrocytes switch their expression profile, upregulate genes involved in osteogenesis and start secreting proangiogenic factors and metalloproteinases (Gawlitta et al., 2010). This promotes blood vessel invasion, the infiltration of osteoprogenitor cells and osteoclasts, and the final remodeling of the cartilaginous template into new bone (Gawlitta et al., 2010).

Over the last decade, tissue engineering has successfully mimicked this process to regenerate bone defects *in vivo* (Thompson et al., 2015). Various types of cells, including multipotent mesenchymal stromal cells (MSCs) (Scotti et al., 2013; Harada et al., 2014; van der Stok et al., 2014; Matsiko et al., 2018; McDermott et al., 2019), embryonic stem cells (Jukes et al., 2008) and adipose-derived stem cells (Osinga et al., 2016) were used alone or in combination with biomaterials to develop a cartilaginous template that, upon implantation, would trigger new bone formation. Despite these promising results, the clinical translation of endochondral bone regeneration (EBR) is in an early stage for several reasons. One of the major challenges is represented by the variability of chondrogenic potential between MSC donors (Gawlitta et al., 2012; van der Stok et al., 2014) and its unpredictability (Sivasubramaniyan et al., 2018). In other words, the successful treatment of all patients with autologous MSCs is not feasible, as the differentiation potential of the isolated MSCs would vary from highly potent to completely incapable, in a patient-dependent manner. Furthermore, the personalized use of cells is associated with high costs when performed under Good Manufacturing Practice (GMP) (Evans et al., 2007; Evans, 2013). Here, we propose the use of non-autologous MSCs (*i.e*., allogeneic or xenogeneic) as a potential strategy to increase the clinical translatability of EBR, ideally in a single-step surgical procedure. Non-autologous cells could be screened and pre-selected for their high chondrogenic potential in advance, circumventing the issue of the donor-to-donor variability, and leading to an off-the-shelf product. In addition, if MSCs with high chondrogenic potential could be pooled and used to treat multiple patients, the high costs associated with isolating and differentiating these cells under GMP restrictions would decrease. Finally, the use of non-autologous MSCs will eliminate any discomfort for the patients related to taking a bone marrow biopsy.

It is evident that the use of allogeneic or xenogeneic cells represents a clinically and economically attractive option. However, the immunogenicity of non-autologous grafts poses a potential obstacle to the clinical implementation, as it could affect the integration and functionality of the grafted tissues (Longoni et al., 2018). Nevertheless, differently from other types of transplantation (e.g., heart, lungs, or liver), in which the grafted organ represents the final functional tissue; in EBR, the cartilage template produced *in vitro* solely serves as a transient substrate that is remodeled into new, mostly recipient-derived bone tissue (Farrell et al., 2011; Scotti et al., 2013). As a result, the host is only gradually and temporarily exposed to the non-autologous MSC-derived chondrocytes and matrix during the remodeling process. Thus, it can be hypothesized that, if the initial remodeling steps would not be hampered by the immune reaction to the engineered non-autologous cartilage, the graft could be replaced by new, partially autologous (Farrell et al., 2011; Scotti et al., 2013) bone tissue. Only a limited number of *in vitro* studies have provided clues about the retention of the MSC immunomodulatory and immunoevasive properties after differentiation. It was shown that allogeneic MSC-derived chondrocytes retain their capability to actively suppress allogeneic T lymphocyte proliferation (Le Blanc et al., 2003; Zheng et al., 2008), decrease the secretion of pro-inflammatory cytokines such as interferon gamma and tumor necrosis factor alpha (Zheng et al., 2008) and inhibit the natural killer cell-mediated cytotoxicity (Du et al., 2016). Additionally, chondrogenically differentiated MSCs do not induce dendritic cell (DC) maturation nor increase in their antigen uptake or migration (Kiernan et al., 2018). On the contrary, it has been reported that xenogeneic, MSC-derived chondrocytes trigger T lymphocyte proliferation, cytotoxicity, and DC maturation, increasing antigen presentation and further activation of the adaptive immune response (Chen et al., 2007). All together, these *in vitro* findings hint that the intensity of the host immune response to the non-autologous implants is different, depending on whether they are allogeneic or xenogeneic. Nevertheless, no study has explored how potential changes in immunological response could affect EBR *in vivo*. As a consequence, based on the available information, it is not possible to predict if in any of these two cases, the host immune response will prevent new bone formation *in vivo*. Therefore, the aim of this study was to evaluate the impact of the immune response evoked by non-autologous MSC-derived chondrocytes on the conversion from cartilage to bone during EBR. To do so, cartilaginous constructs derived from full major histocompatibility complex class I and II (in rats RT1 class I and II) mismatched (Dark Agouti rat) or xenogeneic (human) MSCs were implanted in a critical size femur defect of an immunocompetent rat (Brown Norway) to closely monitor both the elicited immune response and the new bone formation.

## Materials and Methods

### Study Design and Overview

Four experimental groups were included in this study: two different types of a non-autologous cell source, namely allogeneic (Dark Agouti rat, full RT1 mismatch) and xenogeneic (human). These were pre-selected based on their high and similar chondrogenic potential and compared to the syngeneic group (Brown Norway, autologous transplantation). Additionally, a control group consisting of the collagen carrier only was included. A critical size femoral defect introduced in Brown Norway immunocompetent rats was used as a model to observe the immune reaction and EBR induced by the different groups. Two end-points were analyzed, at one (*n* = 5 per group) and 12 weeks (*n* = 8 per group for the syngeneic, allogeneic, and xenogeneic and *n* = 5 for the collagen control group) post-implantation. Mineralization over time was monitored by micro-CT at 0, 4, 8, and 12 weeks after surgery. Systemic immune response was monitored by checking the blood for the presence of an inflammation marker (α-1-acid glycoprotein) and antibody production (IgG and IgM) at 0, 1, 2, 4, 8, and 12 weeks. After euthanasia at 1 or 12 weeks post-implantation, the local immune response was analyzed via immunohistological stainings. Markers belonging to the innate (macrophages: CD68, CD163, iNOS, and CD206) and adaptive (T lymphocytes: CD3) immune response were investigated. Finally, bone formation and remodeling were investigated via histological analysis and histomorphometry (H&E, Safranin-O/Fast Green, and TRAP staining) after 12 weeks.

### Isolation and Expansion of Bone Marrow-Derived MSCs

Human MSCs were isolated from the bone marrow aspirate of a 20-year old female patient. The aspirate was obtained after informed consent, according to a protocol approved by the local Medical Ethics Committee (University Medical Center Utrecht). The mononuclear fraction was separated using Ficoll-paque (Sigma-Aldrich, Zwijndrecht, the Netherlands) and seeded on plastic to select for adherence, as previously described (Gawlitta et al., 2012). The adherent cells were cultured at 37°C under humidified conditions and 5% carbon dioxide (CO_2_) in MSC expansion medium consisting of α-MEM (22561, Invitrogen), supplemented with 10% heat-inactivated fetal bovine serum (S14068S1810, Biowest), 0.2 mM L-ascorbic acid 2-phosphate (A8960, Sigma), 100 U/mL penicillin with 100 mg/mL streptomycin (15140, Invitrogen) and 1 ng/ml basic fibroblast growth factor (233-FB; R&D Systems).

Rat MSCs were isolated from 4 weeks old female Dark Agouti and Brown Norway rats. Briefly, rats were euthanized through CO_2_ asphyxiation and femur and tibia were collected. After removing the epiphysis, bone-marrow was obtained by flushing through the diaphysis with MSC expansion medium supplemented with 0.025% EDTA and cells were plated in a Petri dish. After 24 h, the medium was switched to StemXVivo (CCM004, R&D Systems) and refreshed three times per week. MSCs were passaged at subconfluency until passage 4. MSC multilineage potential (Supplementary Figure 1) was confirmed as reported previously (Gawlitta et al., 2012).

### Chondrogenic Differentiation of MSC Spheroids

At passage 4, human and rat MSCs were harvested for chondrogenic differentiation. Collagen spheroids were created by encapsulating MSCs (20^*^10^6^/ml) into 50 μl collagen type I gel (4 mg/ml) (CB354249, Corning) according to the manufacturer's instruction. Gelation was allowed for 45 min at 37°C. The spheroids were cultured in chondrogenic differentiation medium consisting of high glucose DMEM (31966, Invitrogen) with 1% ITS + premix (354352; BD Biosciences), 10^−7^ M dexamethasone (D8893; Sigma), 0.2 mM L-ascorbic acid 2-phosphate (A8960, Sigma), 100 U/mL penicillin with 100 mg/mL streptomycin (15140, Invitrogen). Chondrogenic differentiation medium for human MSCs was supplemented with 10 ng/ml TGFβ1 (100-21, PeproTech), whereas for rat MSCs, also 100 ng/ml BMP-2 was added. Medium was refreshed every day for the first 4 days and thereafter three times per week. Chondrogenic differentiation was confirmed via histological analysis.

### Construct Preparation

Comparable spheroid sizes were obtained among the different groups after 28 days of differentiation. For each construct, eight chondrogenic spheroids were placed in a square cuboid custom-made mold (3.5 mm × 3.5 mm × 6 mm). Collagen (CB354249, Corning) gels (4 mg/ml) were casted into the mold around the eight spheroids and gelation was allowed for 45 min according to manufacturer's instruction. The constructs were prepared the day before implantation and incubated overnight in chondrogenic differentiation medium without TGFβ1 and BMP-2.

### Animal Experiment and Surgical Procedure

The research protocol and procedures were approved by the animal ethical committee of the University Medical Center Utrecht (2465-2-01) and was in accordance with the national law on animal experiments. Forty-nine male Brown Norway rats (Envigo, the Netherlands) were housed in pairs in the animal facility of the University Medical Center Utrecht. Animals received standard food pellets and water *ad libitum*, under climate-controlled conditions (21°C; 12 h light/12 h darkness). At the age of 12 weeks, after at least 7 days of acclimatization, a 6 mm critical-size segmental bone defect was created under general anesthesia (1–3.5% isoflurane in oxygen, AST Farma, Oudewater, the Netherlands) as previously described (van der Stok et al., 2014). Briefly, the right hind leg was shaved and disinfected. The right femur was exposed through a lateral skin incision and dissection of soft tissue. Three proximal and three distal screws were used to stabilize a 23 × 3 × 2 mm polyether ether ketone (PEEK) plate to the femur in the anterolateral plane. After the bone's fixation to the PEEK plate, a 6 mm bone segment was removed using a saw guide and a wire saw (RISystem, Davos, Switzerland). The collagen constructs were press-fit in the defects and a single dose of antibiotic (Duplocillin LA, 22.000 IE/kg) was injected intramuscularly. Finally, the fascia and skin were sutured in layers using Vicryl Rapide 4-0 (VR 2297, Ethicon). Subcutaneous injection of pain medication (buprenorphine, 0.05 mg/kg bodyweight, AST Farma, Oudewater, the Netherlands) was given pre-operatively and twice a day for the following 3 days. Rats were euthanized after either 1 or 12 weeks with an overdose of barbiturates (phenobarbital; 200 mg/kg body weight, TEVA Pharma, Haarlem, the Netherlands). The femora were analyzed by histology and micro-computed tomography (microCT) scanning.

### MicroCT Scanning

To evaluate tissue mineralization at 0, 4, 8, and 12 weeks after surgery, the hind leg of the rat was fixed in a custom-made support under anesthesia (1–3.5% isoflurane in oxygen) and scanned with a microCT imaging system (Quantum FX; PerkinElmer, Waltham, MA, USA). Three minutes of scanning time was required per leg at an isotropic voxel size of 42 μm resolution (voltage 90 kV, current 180 mA, field of view = 21 mm). All scans were oriented in the same fashion using the ImageJ plugin Reorient3 TP and the same volume of interest (VOI: 6.3 × 5 × 5 mm^3^) was selected for all samples. VOIs were segmented with a global threshold and mineralized volumes were measured in mm^3^ using the image processing software plugin BoneJ (Doube et al., 2010) (Image-J 2.0.0; Java, Redwood Shores, CA, USA). 3D reconstructions of the femur defect were based on the microCT data and created using ParaView (ParaView, Kitware Inc., USA).

### Blood Sampling and Systemic Immune Response

Blood was sampled at 0, 1, 2, 4, 8, and 12 weeks from the tail vein using a catheter (BD angiocath, Becton Dickinson, Vianen, the Netherlands). Serum and plasma were collected in non-coated or EDTA coated MiniCollect tubes (450534 and 450532 Greiner Bio-one), respectively. Sample types were centrifuged for 15 min at 1,500 g and the supernatant was stored at −80°C.

In the serum, the acute-phase protein α-1-acid glycoprotein (AGP), indicative for inflammation, was quantified using an ELISA kit (AGP-2, Life Diagnostic, West Chester, USA), according to the manufacturer's instructions. Total IgG content in the plasma was quantified using the IgG Rat Uncoated ELISA kit (88-50490-86, Invitrogen), according to the manufacturer's instructions.

### Detection of Anti-donor Immunoglobulin in Serum

To assess the production of alloreactive and xenoreactive IgM and IgG by the host, donor MSCs (either syngeneic, allogeneic, or xenogeneic) were expanded until 80% of confluency and chondrogenically differentiated for 10 days (Le Blanc et al., 2003; Chen et al., 2007) in a 96 wells plate. The monolayers were fixed in 10% buffered formalin solution for 30 min and incubated in 5% BSA-PBS for 30 min at room temperature. Rat sera were heat-inactivated for 30 min at 56°C, diluted 1:100 in 5% BSA-PBS and incubated with the donor MSC monolayer correspondent to the type of graft they received *in vivo* for 1 h at room temperature (adapted from Mathieux et al., 2014). After extensive washing with PBS, monolayers were incubated with a TRITC-conjugated antibody [8 μg/ml, goat-anti-rat IgM and IgG (H&L), 3010-03, SouthernBiotech] for 1 h at room temperature. Finally, samples were washed and counterstained with DAPI for 10 min. Representative pictures of the anti-donor immunoglobulin produced 0, 2, and 4 weeks post-implantation were taken for displaying purposes using a confocal microscope (Leica SP8X confocal). At 4 weeks, six pictures per sample (Olympus IX53) taken at random locations were used for the quantification. TRITC pixel quantification and nuclei count were performed using image-J after applying a global threshold. Data are presented as TRITC-positive pixels normalized to the number of nuclei per field of view. Controls for sample cross-reactivity are included in Supplementary Figure 2.

### T Cell Proliferation Assay

To evaluate if the donor cells specifically triggered a T lymphocyte response, at 12 weeks the inguinal and popliteal lymph node were retrieved, crushed, and T cells were stained with CellTrace Violet (C34571, Thermo Fisher) for 20 min at 37°C, according to the manufacturer's instruction. After washing, 2^*^10^5^ stained T cells were resuspended in RPMI (11875093, Invitrogen) supplemented with 10% FBS 100 U/mL penicillin with 100 mg/mL streptomycin and added to the respective donor MSCs, which were beforehand expanded until 80% of confluency and chondrogenically differentiated for 10 days in a 96 wells plate. T cell-donor:MSC co-cultures were incubated for 4 days at 37°C under humidified conditions and 5% CO_2_ (protocol adapted from Ryan et al., 2014). Afterward, cells were detached with trypsin and resuspended in FACS buffer consisting of PBS supplemented with 2% FBS. T cells were stained with CD3-PE conjugated antibody (0.4 μg/ml, 201411, BioLegend, San Diego, USA) for 30 min at 4°C and analyzed on an LSR-Fortessa flow cytometer (BD Bioscience, California, USA). Proliferation peaks were analyzed performing a deconvolution analysis with FlowJo and compared to lymphocytes that were not exposed to any other cell types (Nil) and T lymphocytes co-cultured with a third-party Sprague Dawley MSCs (Aspecific).

### Histology and Immunohistochemistry

At week 1 and 12, the right femora were retrieved for histological processing. All specimens were fixed in a 10% neutral buffered formalin solution for 1 week. After fixation, they were decalcified in a 10% EDTA-phosphate buffered saline solution (pH 7.4), dehydrated in graded ethanol solutions (70–100%) and cleared in xylene. The samples were subsequently embedded in paraffin and sliced into 5 μm thick sections (Microm HM340E). *In vitro* samples were fixed, dehydrated and sliced following a similar procedure. Before staining, samples were deparaffinized with xylene and gradually rehydrated through decreasing ethanol solutions (100–70%).

Overall appearance of sections and new bone formation was evaluated using H&E staining (Sigma). A triple staining of Weigert's hematoxylin (640490; Klinipath BV), fast green (FN1066522; Merck), and Safranin O (FN1164048213; Merck) was applied to identify cell nuclei, collagenous fibers and GAGs. For the TRAP staining, hydrated sections were incubated for 20 min in 0.2 M acetate buffer at room temperature. To identify the osteoclasts, sections were incubated in 0.2 M acetate buffer supplemented with 0.5 mg/ml naphthol AS-MX phosphate (855, Sigma) and 1.1 mg/ml fast red TR salt (F8764, Sigma) for 4 h at 37°C. Mayer's hematoxylin was used for nuclear counterstaining. Histomorphometric analysis was performed on samples stained with H&E. Briefly, an overview of the whole sample was made by merging images (1.25x/0.04 FN26.5 objective) into a panoramic image in Adobe Photoshop C6. For each image, a region of interest (ROI) of 5 × 2.5 mm^2^ was selected in the center of the defect. The titanium screw holes present on each side of the defect were used as reference point in order to ensure an equivalent positioning of ROI in all samples. Three different areas were manually selected for each ROI: bone, hypertrophic cartilage and bone marrow. The amount of pixel for each area was quantified via the function “recording measurement” and normalized for the ROI area. Osteoclasts in the ROI were counted using the cell counter plugin from Image-J.

For collagen type II (II-II6B3), CD68 (ab31630, Abcam), CD206 (AF2535, R&D Systems), CD163 (ab182422, Abcam), CD3 (ab16669, Abcam), and iNOS (ab15323, Abcam) staining, endogenous peroxidase activity was blocked by incubating samples for 15 min with 0.5% H_2_O_2_, followed by aspecific protein blocking with 5% BSA-PBS for 45 min at room temperature. Details about the antigen retrieval, primary and secondary antibody used can be found in Supplementary Table 1. The labels were visualized by DAB oxidation. Sections were then counterstained with hematoxylin, washed, dehydrated and mounted in depex. Rabbit and mouse isotypes (X0903 and X0931, Dako) were used as negative controls at concentrations matched with those of the primary antibodies. All sections were visualized using an Olympus BX51 microscope (Olympus DP73 camera, Olympus). Immune cells were quantified in three different areas of the defect site: the collagen carrier, the infiltrating tissue and the spheroids (Supplementary Figure 3). Three images per sample per area were taken and positive cells were counted using the cell counter plug-in from Image-J. The operator was blinded during the staining, acquisition and counting phases.

### Statistics

The data are presented as means with standard deviations. For the analysis of the microCT results, AGP and IgG levels, a mixed linear model was used. The tests were adjusted for multiple comparisons by a Bonferroni's *post-hoc* comparison test (IBM SPSS 22.0, New York, USA). For the analysis of the immune cells infiltrating the defects (IHC slides) after 1 week and the histomorphometric measures, a Kruskal-Wallis test was performed, followed by a Dunn's *post-hoc* test (GraphPad Prism 6, San Diego, CA, USA). For the analysis of the T cell proliferation a one-way ANOVA was performed, followed by a Dunnet's *post-hoc* test (GraphPad Prism 6). A *p* < 0.05 was considered statistically significant.

## Results

### Macroscopic Observations

At the time of surgery, the mean bodyweight of the rats was 258 ± 27 g and increased to 344 ± 28 g after 12 weeks. In total, three animals (1 xenogeneic, 1 syngeneic, and 1 collagen group) were euthanized before the experimental endpoint was reached due to failure of the PEEK plate and were therefore not included in the analyses. No external signs of adverse reactions (i.e., swelling or redness) to the implants were observed for any of the rats during the course of the experiment.

### *In vitro* Chondrogenic Differentiation

**Figure 1:**
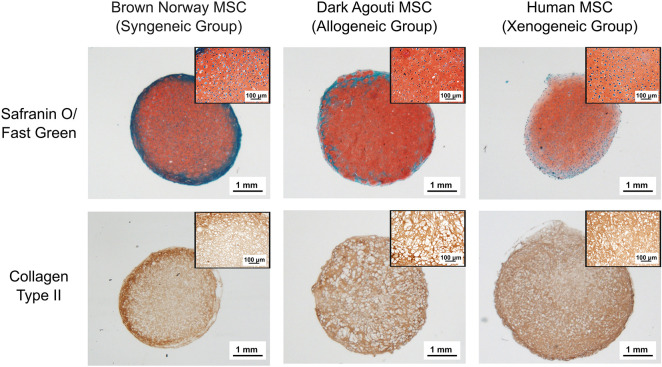
Chondrogenic differentiation of the different MSCs donors. Chondrogenic differentiation is confirmed by the presence of GAGs (red) and Collagen Type II (brown). Inserts contain higher magnification pictures of the collagen spheroids.

After 4 weeks, the collagen spheroids derived from human, Dark Agouti or Brown Norway MSCs displayed abundant glycosaminoglycan (GAG) and collagen type II deposition (Figure 1). Cells displayed the typical chondrocyte morphology, with a rounded shape and were embedded in lacunae.

### New Bone Formation

**Figure 2:**
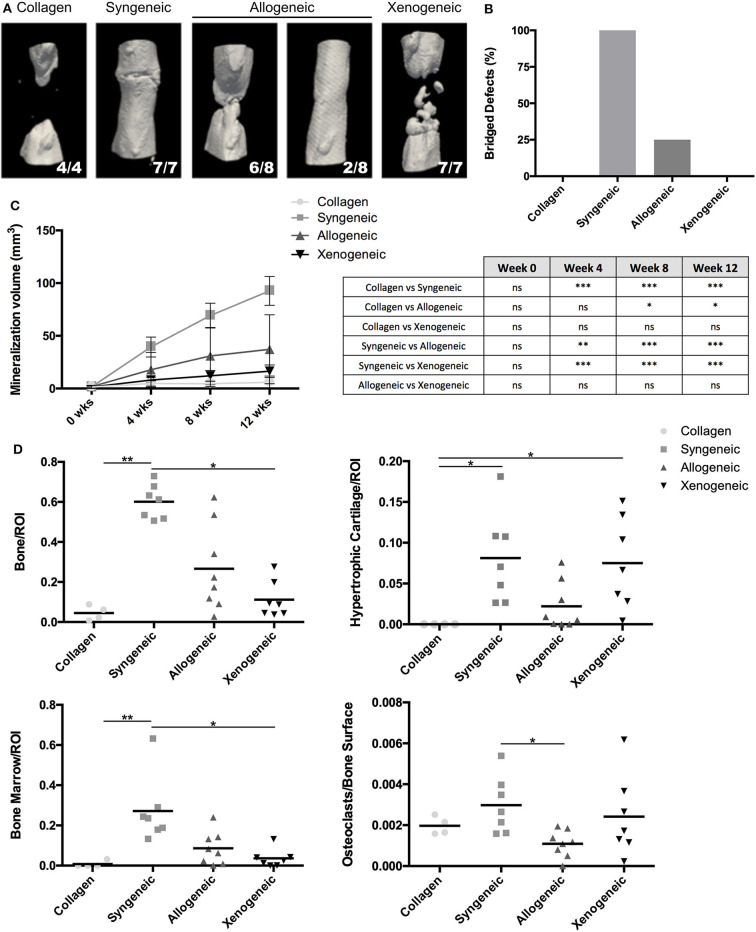
Evaluation of bone formation in the defect area. **(A)** The 3D microCT reconstructions of the defect areas after 12 weeks highlight the presence of mineralized areas with the shape of the implanted cartilaginous spheroids in the allogeneic and xenogeneic groups. **(B)** Heterogeneous results were observed in the allogeneic group, with 2/8 full bridging of the defect (25%) whereas 7/7 defects were bridged in the syngeneic group (100%). **(C)** Quantification of the mineralization over time confirmed that new bone formation was enhanced in the syngeneic group. **(D)** Results of the histomorphometric analysis performed 12 weeks post-implantation presented a similar trend. *p < 0.05; **p < 0.01; and ***p < 0.001. ns: not significant.

After 12 weeks, new bone formation was observed close to the extremities of the osteotomy gap in all samples. 7/7 defects of the syngeneic control group and 2/8 of the allogeneic group were fully bridged whereas no full bridges were observed in the xenogeneic or the collagen groups (Figures 2A,B). Furthermore, bone regeneration in the center of the defect was observed in all the samples in which MSCs were implanted, but not in the collagen control (Figure 2A). In particular, mineralized volumes resembling the shape and size of the implanted spheroid structures were still evident, especially in the xenogeneic group (Figure 2A). In these areas, human cells were found in the newly formed bone, suggesting the direct involvement of the cartilaginous templates in the regenerative process (Supplementary Figure 4).

**Figure 3:**
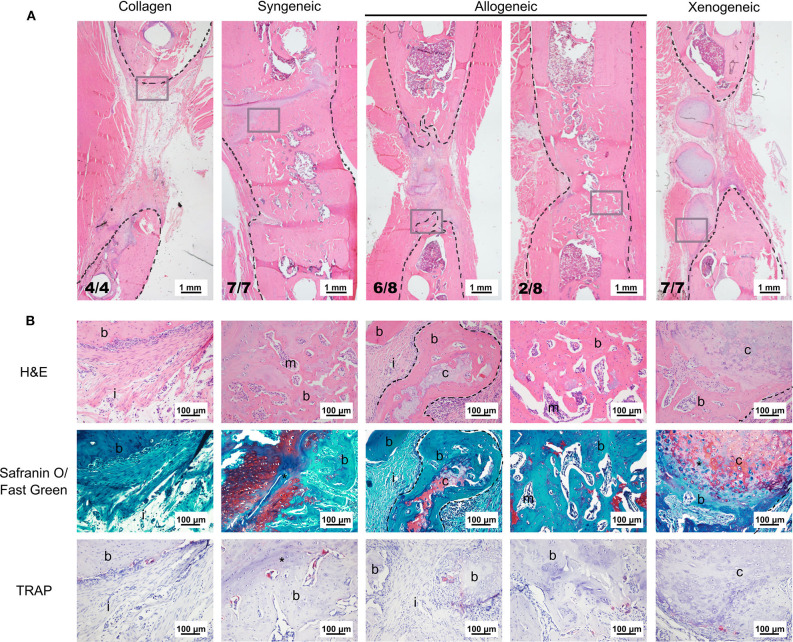
Representative images of the bone defect repair after 12 weeks. **(A)** Overview of the defect area stained with H&E. The black dotted lines indicate the bone edges whereas the gray boxes highlight the area depicted in the higher magnification in **(B)**. **(B)** H&E staining shows that the edges of the cartilaginous spheroids (limited by the dotted line) were converted into bone. Safranin O/fast green staining highlights active EBR in the syngeneic group and the presence of some non-remodeled cartilage (red) in the core of the spheroids of the allogeneic and xenogeneic groups. TRAP staining (bright pink) indicates the presence of active osteoclasts in all groups. i, tissue infiltration; b, bone; m, bone marrow; c, cartilaginous spheroids; *EBR.

Based on microCT data, mineralization was highest in the syngeneic group (92.6 ± 13.7 mm^3^), followed by 37.2 ± 32.6 mm^3^ for allogeneic, 16.4 ± 5.9 mm^3^ for xenogeneic, and 5.96 ± 5.9 mm^3^ for the collagen control (Figure 2C). A similar trend was observed after histomorphometric analysis (Figure 2D). Interestingly, hypertrophic cartilage was present also in the syngeneic group, indicating that remodeling is still ongoing at the proximal edge of the defect. On the contrary, in the allogeneic and xenogeneic group the hypertrophic cartilage was predominantly found in the non-remodeled parts of the spheroids. Consistent with the 3D reconstructions, the H&E staining highlighted the presence of bone islands at the edges of the spheroidal structures (Figure 3). Finally, in areas of active EBR, it was possible to discern osteoclasts, confirming that remodeling was ongoing after 12 weeks (Figures 2D, 3B).

### Implant-Induced Early Macrophage Polarization and T-Cell Infiltration

**Figure 4:**
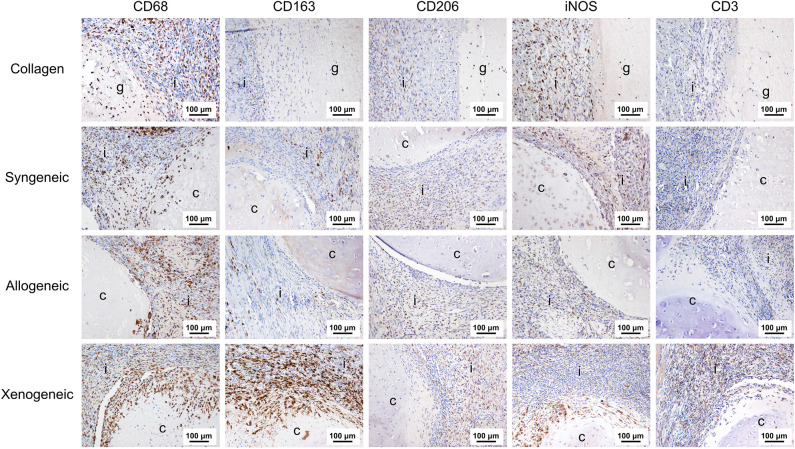
Local adaptive and innate immune response after 1 week from the implantation. CD68+ and CD163+ macrophages were particularly abundant in the xenogeneic group, especially in the spheroids infiltration. No differences were evident in CD206+ macrophages distribution whereas more iNOS + cells were infiltrating the cartilaginous spheroids. CD3+ lymphocytes were predominant in the osteotomy gap of the xenogeneic samples compared to the other groups. Brown color indicates the positive staining. i, tissue infiltration; c, cartilaginous spheroids; g, collagen gel.

**Figure 5:**
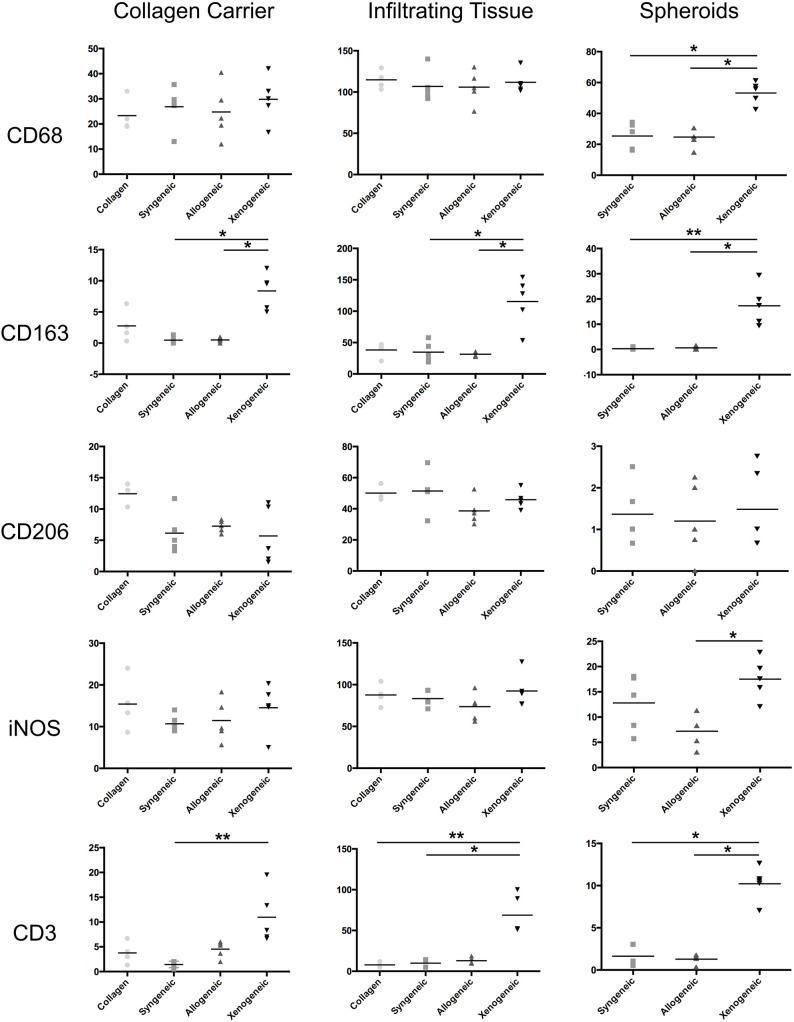
Quantification of the immune cell infiltration in the different defect areas (collagen remnant, infiltration, and spheroids) after 1 week. The major differences were observed in the spheroids, where significantly more CD68+ and CD163+ macrophages, iNOS+ cells and CD3+ lymphocytes were found in the xenogeneic group. *p < 0.05; **p < 0.01.

One week after implantation, macrophages positive for CD68 were most abundant in the xenogeneic group, especially when considering the tissue infiltration in the implanted spheroids (Figures 4, 5). Similarly, CD163+ macrophages were most abundant in the xenogeneic group in all the three analyzed areas (Figures 4, 5). When analyzing the polarization of the macrophages, a significantly higher (*p*-value 0.01) amount of pro-inflammatory iNOS+ cells was detected in the spheroids of the xenogeneic group whereas no differences in anti-inflammatory CD206+ macrophages were observed (Figure 5). Finally, CD3+ lymphocytes were higher for the xenogeneic group in all three analyzed areas; the collagen carrier, the infiltrating tissue and the spheroids (Figures 4, 5). On the contrary, no differences in T-cell infiltration or macrophage polarization were observed between the collagen, syngeneic and allogeneic groups.

### Late T Cell Response

**Figure 6:**
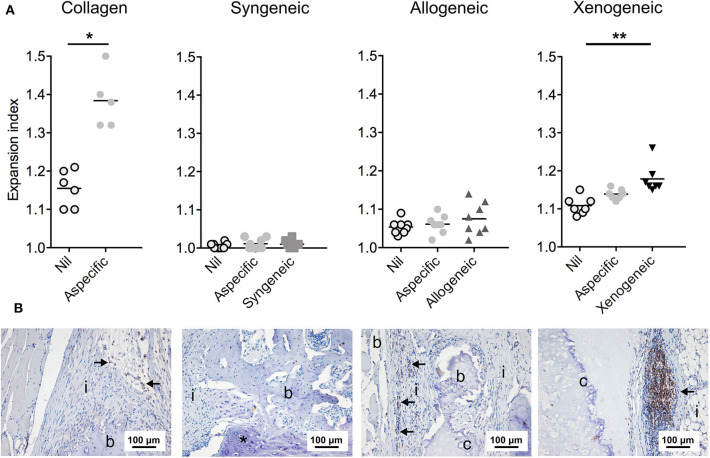
T lymphocyte reaction to the non-autologous implants after 12 weeks. **(A)** Proliferation of the T cells isolated from the draining lymph nodes was assessed in a co-culture model with the donor cells. Specific proliferation induced by the donor cells was observed only in the xenogeneic group. Nil: lymphocytes that were not exposed to any other cell type; Aspecific: lymphocytes co-cultured with third-party Sprague Dawley MSCs; *p < 0.05; **p < 0.01. **(B)** CD3+ T lymphocytes (indicated by the arrows) were still prominent in the local immune response against the xenogeneic implants. Fewer CD3+ cells were spotted in the collagen and allogeneic samples. Brown color indicates the positive staining; i, tissue infiltration; b, bone; c, cartilaginous construct; *EBR.

Only the chondrogenically differentiated donor MSCs form the xenogeneic group stimulated the proliferation of T-cells isolated from the draining lymph nodes adjacent to the implant during the *in vitro* co-culturing with donor cells (Figure 6A). Accordingly, the xenogeneic group presented the highest level of T-lymphocyte infiltration *in vivo*, after 12 weeks from implantation (Figure 6B). No T-cell proliferation was observed in the co-culture model for the allogeneic and the syngeneic group. Consistently, a limited amount of CD3+ lymphocytes was present in the defect after 12 weeks.

### Systemic Immune Response and Antibody Production

**Figure 7:**
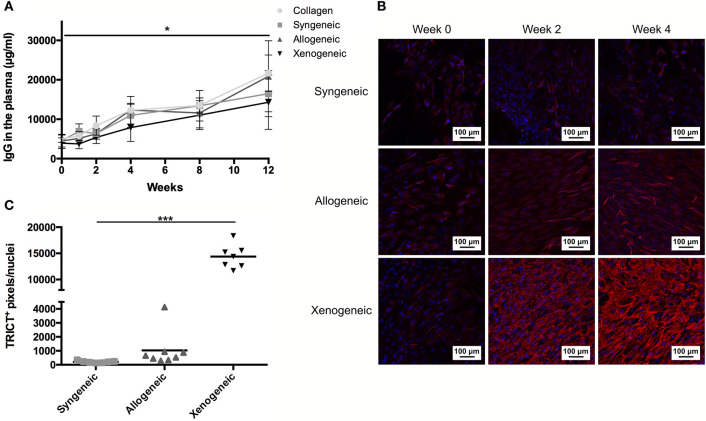
Humoral response to the non-autologous implants. **(A)** Systemic IgG quantification shows a significant increase over time for all samples, but no differences between groups at any time-points. **(B)** Specific anti-donor IgG and IgM (red staining) were observed in all groups, but in particular in the xenogeneic group. **(C)** Quantification of the fluorescent staining confirmed that significantly more antibodies are produced against the xenogeneic implant. Although not statistically significant, more antibodies are produced on average against the allogeneic implants compared to the syngeneic ones. *p < 0.05 and ***p < 0.001.

An increase in serum concentration of AGP, an acute phase protein produced by the liver, was observed for all groups 1 week after the surgery (Supplementary Figure 5) as a consequence of the tissue injury during surgery. However, the AGP level had returned to baseline values in all animals after 2 weeks. On the contrary, the total IgG concentration in the plasma continued to increase over the 12 weeks, without any statistically significant differences between groups at any of the analyzed time-points (Figure 7A). However, when analyzing the binding of IgM and IgG to the implanted MSCs by immunocytochemistry, differences were observed (Figures 7B,C). In particular, lower levels of specific anti-donor immunoglobulins were detected in the syngeneic group (mean of 213 ± 74 positive pixels/nuclei) compared to both, the xenogeneic (mean of 14,382 ± 2,259 positive pixels/nuclei) and the allogeneic groups (mean of 1,032 ± 1,276 positive pixels/nuclei).

## Discussion

The use of non-autologous MSCs with high chondrogenic differentiation capability has the potential to open up new avenues for the clinical translation of cell-based methods for EBR. For such a therapy to be viable, it is crucial to unravel if, and to which extent, the host immune reaction against the foreign implants prevents new bone formation. Here, using immunocompetent animals as a model, we proved that the conversion of the xenogeneic or allogeneic cartilaginous substrates into new bone is feasible even in the presence of a functional immune system. However, the extent of tissue mineralization was found to increase as a function of how close the donor cells are related to the recipient. This suggests that the activation of the immune system played a role in hampering EBR.

Xenogeneic cartilaginous spheroids suffered from an immune rejection, which impaired bone healing. Interestingly, despite this rejection, bone formation was not entirely inhibited in this group. The strong immune response was mediated by multiple immune cell types, belonging to both the innate and adaptive branch of the immune system. In particular, CD68+ and CD163+ macrophages were significantly more present within and in the proximity of the xenogeneic spheroids. This is in line with previous observations, where macrophages were found to be one of the driving forces responsible for the rejection of xenogeneic primary chondrocytes in an orthotopic minipig model (Niemietz et al., 2014). The direct involvement of macrophages in xenotransplantation rejection is probably due to their intrinsic capability of identifying non-self-cells, through the recognition of species-specific surface antigens such as CD47 (Ide et al., 2007; Navarro-Alvarez and Yang, 2014). The expression of iNOS in the majority of the cells that infiltrated the xenogeneic spheroids further supports their involvement in the cartilaginous spheroid rejection, as iNOS is a marker that usually indicates macrophage polarization toward a pro-inflammatory phenotype. Furthermore, the presence of iNOS positive macrophages usually correlates with poor regenerative outcomes (Brown et al., 2009). B and T lymphocytes were also involved in the rejection of the xenogeneic implants. In particular, CD3+ T lymphocytes infiltrated both the implanted construct and the surrounding tissue already within 1 week, and their presence persisted until the explantation at 12 weeks. This could have negatively affected the remodeling of the cartilaginous constructs, as T lymphocytes can promote the lysis of the grafted cells and stimulate the activation of other immune cells, including macrophages and B lymphocytes (Moreau et al., 2013). In addition, a correlation between the prolonged presence of effector T cells and delayed fracture repair was previously established (Reinke et al., 2013; Kovach et al., 2015; Schlundt et al., 2019). We further confirmed that the xenogeneic antigens specifically activated host lymphocytes, as in the co-culture model chondrogenically differentiated human MSCs induced the proliferation of T cells isolated from the draining lymph node. Similarly, by exposing the rat serum to human MSC-derived chondrocytes, the presence of antibodies against the xenogeneic antigens was observed. These findings are in line with the activation pattern of the adaptive branch of the immune system during both acute and chronic transplant rejection (Grinnemo et al., 2004; Mathieux et al., 2014; Vadori and Cozzi, 2015).

In contrast to the xenogeneic group, only a limited immune response was observed in the allogeneic group. More specifically, 1 week post-implantation no differences between the syngeneic and the allogeneic group were found in terms of CD68+ and CD163+ macrophage infiltration and M1 (iNOS)/M2 (CD206) polarization in the defect area and in the cartilaginous spheroids. Similarly, when comparing CD3+ T cell infiltration within the engineered constructs and in the surrounding tissues *in vivo*, no differences between the allogeneic and the syngeneic groups were observed at both 1 and 12 weeks post-implantation. Furthermore, no T cell proliferation was induced *in vitro* after co-culture with allogeneic MSCs. This outcome reinforces previous *in vitro* findings that suggest retention of immunoevasive or immunomodulatory properties in allogeneic chondrogenically differentiated MSCs (Le Blanc et al., 2003; Kiernan et al., 2016, 2017). To the best of our knowledge, only two other studies analyzed the immune response elicited by allogeneic MSCs-derived chondrocytes *in vivo* albeit for cartilage TE applications (Butnariu-Ephrat et al., 1996; Ryan et al., 2014). Here, chondrogenically differentiated MSCs were encapsulated in an alginate (Ryan et al., 2014) or hyaluronic acid carrier (Butnariu-Ephrat et al., 1996) and implanted subcutaneously (Ryan et al., 2014) or in an articular cartilage defect (Butnariu-Ephrat et al., 1996). CD68+ macrophage and CD3+ lymphocyte infiltration (Ryan et al., 2014) and fibrosis (Butnariu-Ephrat et al., 1996) were reported after 6 (Ryan et al., 2014) and 12 (Butnariu-Ephrat et al., 1996) weeks, respectively. Based on our findings, such an exacerbated immune response was not stimulated upon implantation in a segmental bone defect. Furthermore, differently from these studies, which aimed at obtaining stable cartilage, the unique goal of our study was to exploit the allogeneic spheroids only as a temporary substrate to trigger EBR. Thus, at the 12 weeks mark, the allogeneic graft was partially or entirely remodeled into new, partially autologous bone tissue. As a consequence, it is possible that this gradual remodeling over time and the cartilage conversion to non-immunogenic host neo-tissue did not trigger any additional immune cell activation and migration. Nevertheless, in spite of an immune reaction comparable to the syngeneic control in terms of early inflammation, macrophage and T lymphocyte infiltration and activation, the extent of bone formation showed more variability across the animals within the allogeneic group. In particular, 2/8 animals displayed full regeneration, comparable to the one induced by the syngeneic constructs whereas in 6/8 rats only partial regeneration was observed. While the origin of this difference could be multifaceted, one important contribution could be the production of IgM and IgG by the B lymphocytes. A trend toward increased IgM and IgG production against the allogeneic implant was observed after 4 weeks. Production of alloreactive antibodies against different antigens, including the major histocompatibility complex (MHC) class I and class II molecules, the ABO blood-group antigens and other minor alloantigens, has been reported in several preclinical models (Colvin and Smith, 2005; Ryan et al., 2014; Lohan et al., 2017), marking the pivotal role of B cells in allorejection. In our study, the presence of immunoglobulin might have induced the activation of the complement system (Colvin and Smith, 2005; Su et al., 2019), interfering with the total remodeling of the construct in a host-dependent fashion. In particular, it is known that receptors for the complement anaphylatoxins (e.g., C5aR and C3aR) are expressed not only by immune cells, but also by cells involved in the fracture repair like osteoblasts, hypertrophic chondroblasts and osteoclasts (Huber-Lang et al., 2013; Mödinger et al., 2018). In addition, altering their expression pattern was shown to alter of the inflammatory phase of fracture healing and ultimately impaired bone repair (Bergdolt et al., 2017; Mödinger et al., 2018). Thus, future studies in the field could unravel if transient suppression of the B cell response would allow a more homogeneous and predictable extent of bone formation from allogeneic engineered cartilage grafts. Furthermore, investigating the role played by other immune cells, including polymorphonuclear cells, natural killer cells and the complement system could shed the light on additional reasons behind the heterogeneous outcome observed in the allogeneic group.

Importantly, in our study we explored the feasibility of EBR in the context of a full RT1 class I and II allogeneic mismatch (Gill et al., 1987). Nevertheless, even in such a challenging immune mismatch, successful bone regeneration comparable to the one induced by the syngeneic grafts was observed in 25% of the cases. These results present first hints toward a potential clinical translation, provided that a more homogeneous and predictable outcome could be achieved. An interesting option that could be explored is a partial donor-recipient MHC match. A partial RT 1 match might decrease the alloantibody production and promote a more reliable regenerative outcome, opening the way to a fully reproducible protocol for optimizing allogeneic EBR-based strategies.

On a final note, controversial evidence exists in literature regarding the direct contribution of MSCs to tissue regeneration. In particular, MSC secretome also exhibits regenerative capacity, as it promotes immune modulation, cell survival and reduces tissue fibrosis (Spees et al., 2016). Nevertheless, it has been established in several studies that in EBR, the implanted cells directly contribute to new bone formation, as part of the non-autologous chondrocytes transdifferentiate toward osteoblasts or osteocytes, and persist in the implanted matrix (Farrell et al., 2011; Scotti et al., 2013; Bahney et al., 2014; Yang et al., 2014). Thus, it must be considered that the newly formed tissue could contain donor cells and this might still affect bone homeostasis at a later stage, as an immune system reactivation could damage the newly formed bone. Although further analyses are required to exclude this possibility, our results suggest that the rejection of the newly deposited tissue is not a likely event. Our histological analyses do not show any sign of degradation of the newly formed bone after 12 weeks, underlining the safety and feasibility of using allogeneic cell sources for EBR.

## Conclusion

The use of non-autologous MSCs for EBR offers great benefits from a translational clinical perspective, such as enabling a pre-selection of MSCs with high chondrogenic differentiation potential to guarantee a beneficial therapeutic outcome. Our results represent the first proof-of-concept of the feasibility of using non-autologous, chondrogenically differentiated MSCs to trigger EBR. A severe immune response did result in a low level of bone formation in the xenogeneic group, rendering it unsuitable for clinical translation applications. On the contrary, a milder immune response, mainly characterized by the production of specific anti-donor IgM and IgG was observed in the allogeneic group. While this might have affected the variability in terms of percentage of defect bridging between the different experimental animals, the successful bone formation observed in the allogeneic group provides encouraging evidence of its potential as an alternative to autologous transplantation. Overall, these findings provide fundamental information for the design and translation of the next generation of EBR-based strategies.

## Data Availability Statement

All datasets generated for this study are included in the article/Supplementary Material.

## Ethics Statement

This animal study was reviewed and approved by Animal Welfare Body Utrecht (approved work Protocol No. 2465-2-01).

## Author Contributions

AL: conception and design, collection and assembly of the data, data analysis and interpretation, manuscript writing, and final approval of the manuscript. IP: collection and assembly of the data, data analysis and interpretation, and final approval of the manuscript. MC: conception and design, collection and assembly of the data, data analysis and interpretation, and final approval of the manuscript. MR, VP, and RL: data analysis and interpretation and final approval of the manuscript. AR: financial support, data analysis and interpretation, and final approval of the manuscript. DG: conception and design, data analysis and interpretation, financial and administrative support, and final approval of the manuscript. All authors contributed to the article and approved the submitted version.

## Conflict of Interest

The authors declare that the research was conducted in the absence of any commercial or financial relationships that could be construed as a potential conflict of interest.
