# Supplementary material for: Endochondral Bone Regeneration by Non-autologous Mesenchymal Stem Cells
Source: Front Bioeng Biotechnol. 2020 Jul 9;8:651. doi: 10.3389/fbioe.2020.00651 (PMC7363768; doi:10.3389/fbioe.2020.00651)
Supplement: Supplementary file 1 [file Table_1.docx]

Supplementary Material

# Supplementary Table 1. Specifics of the antigen retrieval method, and primary and secondary antibodies used for the immunohistochemical analyses.

| Primary antibody | Concentration | Antigen retrieval | Secondary antibody |
| --- | --- | --- | --- |
| Collagen type II | 0.6 μg/ml, incubation O/N at 4°C | Sequential incubation with 1mg/ml pronase and 1mg/ml hyaluronidase at 37° for 30m | BrightVision poly HRP-anti-mouse IgG (VWRKDPVM110HRP) |
| CD68 | 4 μg/ml, incubation O/N at 4° C | Incubation with 0.1% pepsin at 37° for 30m | BrightVision poly HRP-anti-mouse IgG (VWRKDPVM110HRP) |
| CD206 | 3.3 μg/ml incubation O/N at 4° C | Boiling at 80° in 10mM citrate buffer pH 6 for 45m | Donkey anti-Goat IgG H&L (ab6886) |
| CD163 | 1.342 μg/ml incubation O/N at 4° C | Boiling at 95° in TRIS-EDTA buffer pH 9 for 20m | BrightVision poly HRP-anti-rabbit IgG (VWRKDPVR110HRP) |
| CD3 | 20 μg/ml incubation O/N at 4° C | Boiling at 95° in 10mM citrate buffer pH 6 for 20m | BrightVision poly HRP-anti-rabbit IgG (VWRKDPVR110HRP) |
| iNOS | 0.52 μg/ml incubation O/N at 4° C | Boiling at 80° in 10mM citrate buffer pH 6 for 45m | BrightVision poly HRP-anti-rabbit IgG (VWRKDPVR110HRP) |
| human mitochondria (ab92824) | 1 μg/ml incubation O/N at 4° | Boiling at 95° in TRIS-EDTA buffer pH 9 for 30m | BrightVision poly HRP-anti-mouse IgG (VWRKDPVM110HRP) |

## Supplementary Figures:


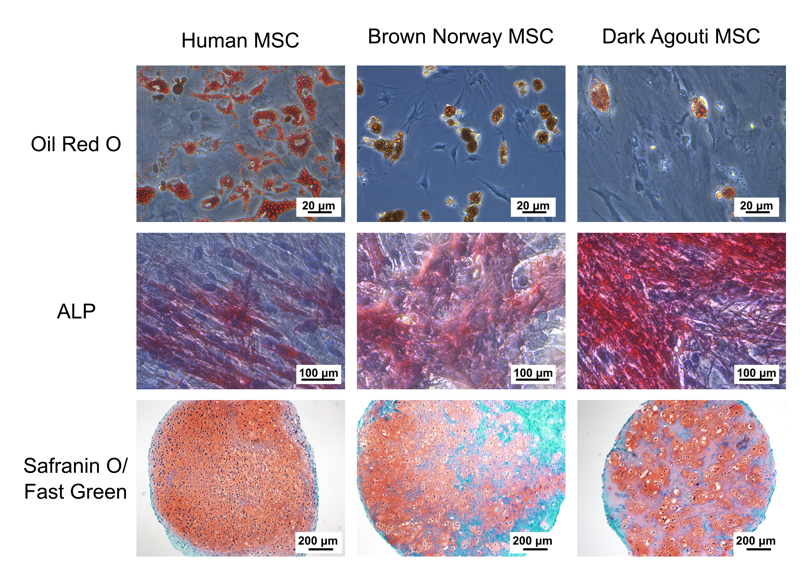


**Supplementary Figure 1.** Three lineage differentiation of the isolated MSCs. The differentiation towards the adipogenic (oil red O), osteogenic (alkaline phosphatase, ALP) and chondrogenic (Safranin O/ Fast green) lineage confirm the multipotency of the isolated MSCs.


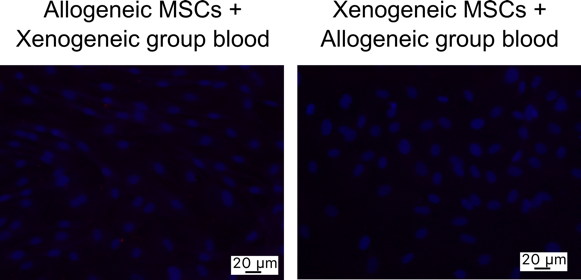


Supplementary Figure 2. Representative images of the cross-controls for results shown in Figure 7. For each condition, three different serum samples, in which a positive signal was detected when exposed to their respective group, were incubated with the cells from a different group. No TRITC-positive staining was observed.


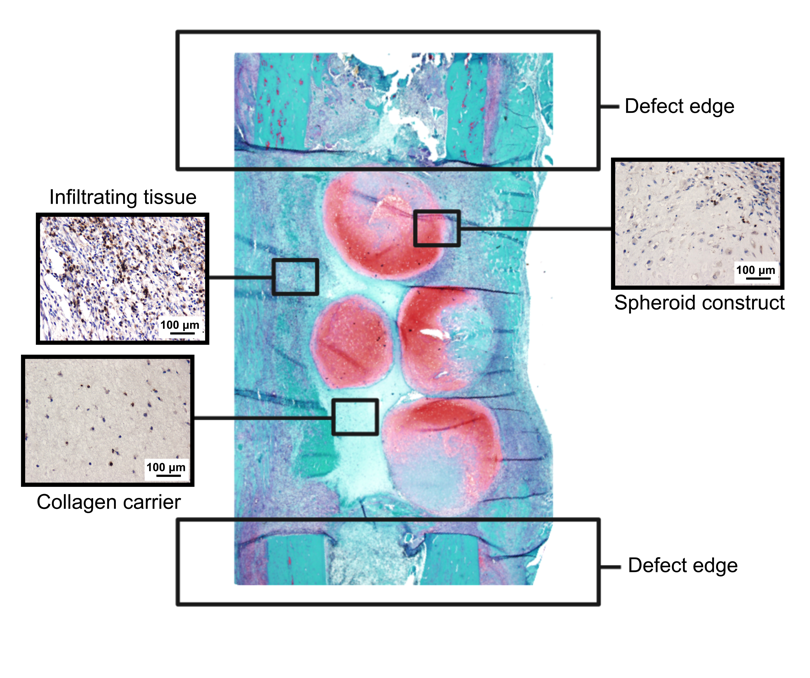


**Supplementary Figure 3.** Overview of the defect (Safranin O/fast green staining) and the three areas selected for the immune cell quantification after 1 week. Examples of images used for the CD3+ lymphocyte quantification are shown.


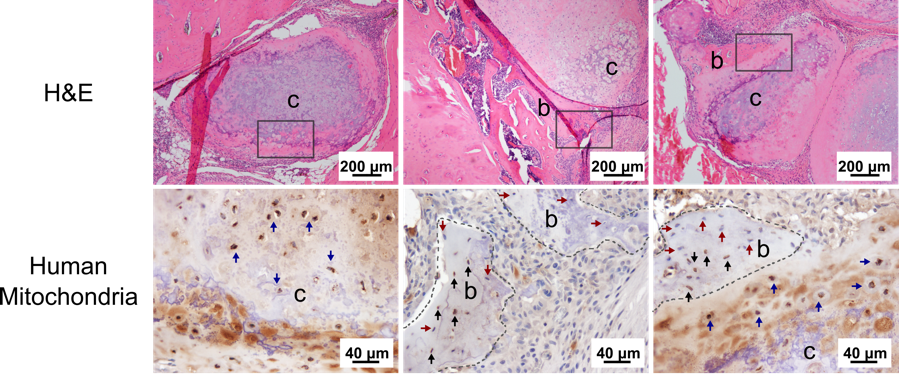


Supplementary Figure 4. Anti-human mitochondria staining. Human cells were still found 12 weeks post-implantation within the cartilage spheroids in several samples of the xenogeneic group (brown intra-cellular staining). In addition, human cells seem to directly contribute the tissue regeneration, as they were found in the mineralized cartilage (blue arrows) and in areas of the spheroids where cartilage had been converted into new bone (black arrows). The newly formed bone contains both human and rats cells (the latter are indicated by red arrows). b: bone, c: cartilage, dark grey box: area depicted in the anti-mitochondria staining, light grey dotted line: outline of bone tissue.


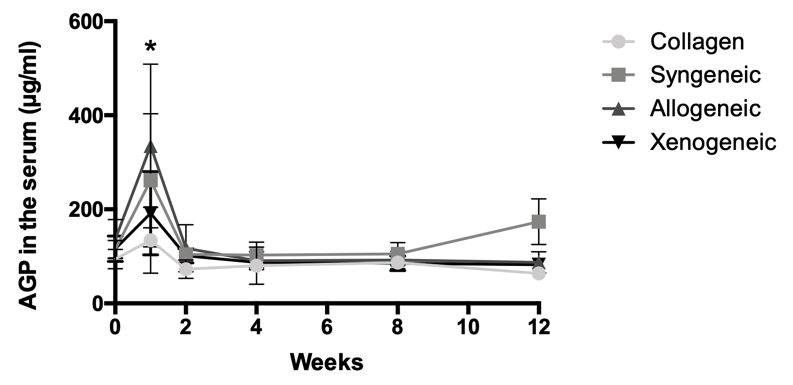


**Supplementary Figure 5.** α-1-acid glycoprotein (AGP) quantification in the serum of the rats of the four groups. No differences were observed between groups over time, except for an increase of AGP concentration in all groups after 1 week, most probably due to the surgery. **p*<0.05
